# The components of self-rated health among adults in Ouagadougou, Burkina Faso

**DOI:** 10.1186/1478-7954-11-15

**Published:** 2013-08-08

**Authors:** Yentéma Onadja, Simona Bignami, Clémentine Rossier, Maria-Victoria Zunzunegui

**Affiliations:** 1 Département de démographie, Université de Montréal, 3150 rue Jean-Brillant, local C-5043, H3T 1N8 Montréal (Québec), Canada; 2 Institut Supérieur des Sciences de la Population (ISSP), Université de Ouagadougou, 03 BP 7118, Ouagadougou 03, Burkina Faso; 3 Institut national d’études démographiques (INED), 133 Boulevard Davout, 75980 Paris Cédex 20, France; 4 Département de médecine sociale et préventive, Université de Montréal, 1420 Mont-Royal, H2V 4P3 Montréal (Québec), Canada; 5 Centre de Recherche du Centre Hospitalier de l’Université de Montréal (CRCHUM), Université de Montréal, 3875 rue Saint Urbain, 5e étage, H2W 1V1 Montréal (Québec), Canada

**Keywords:** Ouagadougou, Burkina Faso, Self-rated health, Chronic diseases, Functional limitations, Depression, Adults

## Abstract

**Background:**

Although the relationship between self-rated health (SRH) and physical and mental health is well documented in developed countries, very few studies have analyzed this association in the developing world, particularly in Africa. In this study, we examine the associations of SRH with measures of physical and mental health (chronic diseases, functional limitations, and depression) among adults in Ouagadougou, Burkina Faso, and how these associations vary by sex, age, and education level.

**Methods:**

This study was based on 2195 individuals aged 15 years or older who participated in a cross-sectional interviewer-administered health survey conducted in 2010 in areas of the Ouagadougou Health and Demographic Surveillance System. Logistic regression models were used to analyze the associations of poor SRH with chronic diseases, functional limitations, and depression, first in the whole sample and then stratified by sex, age, and education level.

**Results:**

Poor SRH was strongly correlated with chronic diseases and functional limitations, but not with depression, suggesting that in this context, physical health probably makes up most of people’s perceptions of their health status. The effect of functional limitations on poor SRH increased with age, probably because the ability to circumvent or compensate for a disability diminishes with age. The effect of functional limitations was also stronger among the least educated, probably because physical integrity is more important for people who depend on it for their livelihood. In contrast, the effect of chronic diseases appeared to decrease with age. No variation by sex was observed in the associations of SRH with chronic diseases, functional limitations, or depression.

**Conclusions:**

Our findings suggest that different subpopulations delineated by age and education level weight the components of health differently in their self-rated health in Ouagadougou, Burkina Faso. In-depth studies are needed to understand why and how these groups do so.

## Background

Self-rated health (SRH), generally captured by a single item inviting respondents to provide an overall assessment of their health using some form of a five-point scale (for example, very good, good, fair, poor, and very poor), is currently one of the most commonly used health measures in surveys to assess the health status of adult populations in developed countries. In these countries, despite its many limitations [1], this indicator has become increasingly popular for assessing health status because of its simplicity and its solid well-established links with various health indicators such as mortality [2,3], functional limitations [4,5], and chronic diseases [6,7].

At the theoretical level, it has been assumed that SRH is a good predictor of mortality because it is an overall measure that incorporates several dimensions of health [8]. Empirically, quantitative studies conducted in developed countries have examined this multidimensional nature and found a strong correlation between SRH and a wide range of measures of physical and mental health [9-13]. Supporting the results of these quantitative studies, several qualitative studies carried out in developed countries have revealed that the different components of health, both serious and less serious, made up the core nucleus of factors underlying SRH [14-17].

However, little information is available on the multidimensional nature of SRH in developing countries. The studies that have examined this issue have focused mainly on Asian countries and on the elderly [18,19]. With regard to SRH in the African context, the knowledge is rudimentary and very incomplete. Existing studies have focused on the social determinants of SRH in African countries [20-25], rather than on the measures of physical and mental health that make up SRH. In addition, with a few exceptions [20,23], most of these studies have focused on specific age groups, either adolescents [25], younger adults [21], or older adults [22,24], making it difficult to adequately analyze the modifying effects of age on the relationship between SRH and the physical and mental dimensions of health.

Indeed, research suggests that age is one of the most important socio-demographic factors affecting both what potential components of health a person considers and how they are taken into account in SRH [8]. Thus, according to social comparison theory, older people have lower expectations regarding health than do younger people [8], and these expectations can lead to more positive assessments among the elderly and more negative assessments in the young [11]. In developed countries, social comparison theory has received a certain amount of empirical support [11], which has found that the relationship between poor SRH, on one hand, and chronic diseases and functional limitations, on the other, diminished with age.

Sex is another key variable that can modify the relationship between SRH and physical and mental health. Compared to men, women have been found to be less “stoic” and thus more likely to take less serious illnesses into account when assessing their health [26]. Likewise, according to Iburg and colleagues [27], “the frequently observed pattern in many health surveys in which women report worse health than men may therefore be understood not simply as an indicator of lower health levels, but of higher expectations for health” (p. 14). Nonetheless, Case and Paxson [28] showed that, even if women more often report worse health than men, women and men with the same chronic conditions have the same SRH. These authors explained that sex differences in SRH could be entirely explained by differences in the distribution of chronic conditions. Similarly, other authors [9,29] have found only a very marginal impact of sex on the relationship between SRH and physical and mental health.

A third factor that may modify the relationship between SRH and physical and mental health is education. For example, recent studies in the United States and France have found that the effects of chronic diseases and of functional limitations on SRH were stronger among more educated individuals [30-32]. One hypothesis to explain this apparent paradox might be that rising levels of education may be accompanied by greater health expectations, and consequently people with more education may assess their health more negatively than those who are less educated for the same health problems [32]. This idea was not supported by Smith and colleagues [13], however, who showed that the effects of chronic conditions, functional limitations, and some aspects of mental health on SRH were similar across education levels in Canada.

In summary, although the relationship between SRH and measures of physical and mental health is well-documented in developed countries, very few studies have examined this association in the developing world, particularly in Africa. In addition, the contradictory results about the modifying effects of sex, age, and educational level stress how social heterogeneity in reporting perceived health depends on the cultural context [33] and highlight the importance of studying this issue in African settings.

This study attempts to fill these gaps by using data from the 2010 Health Survey carried out at the Ouagadougou Health and Demographic Surveillance System (Ouaga HDSS) to examine the associations between SRH and measures of physical and mental health (chronic diseases, functional limitations, depression) among adults 15 years or older in Ouagadougou, Burkina Faso, and how these associations vary by sex, age, and education level. This survey is currently the most up-to-date source of data purposefully designed to obtain information on adult health and health determinants in an urban environment in Burkina Faso.

### Study setting

As in other large African cities, Ouagadougou’s demographic structure is characterized by a high proportion of young persons. While 39% of the population of Ouagadougou in 1996 and 36% in 2006 was below the age of 15 years, persons 65 years or older represented only 1.8% and 1.9% of the population in those years, respectively [34]. Between 1996 and 2006, Ouagadougou registered an annual demographic growth rate of 4.2%, half of which was attributed to immigration of rural youth seeking socio-economic opportunities [35]. In addition, between 1998 and 2010, Ouagadougou’s fertility rate went from 4.1 to 3.4 children per woman [36,37]. With respect to overall mortality, although there are no specific data for Ouagadougou, we can say it has considerably declined, especially given that life expectancy at birth rose from 56.3 years in 1996 to 64.3 years in 2006 in urban areas [38]. As for the socio-economic status of the population, 38% of persons aged 6 years or older were unschooled in 2006 in Ouagadougou [39]. That proportion was higher for women (44%) than for men (33%). In addition, in 2009, more than 30% of the population lived in informal settlements in Ouagadougou’s peripheral zones [40], with a very limited supply of infrastructure and public services [41]. However, in the areas covered by the Ouaga HDSS, the levels of social support (neighbors, friends, professional relationships, associations) are high [42]. From an epidemiological standpoint, Ouagadougou’s population, like those in the other sub-Saharan African regions, is still burdened with infectious diseases, particularly malaria, which continues to be the primary cause of death. Nevertheless, in recent years, chronic conditions such as hypertension [43], visual difficulties, and blindness [44] have been steadily rising in the African context. Obesity is also on the rise in West African adult populations [45], and Ouagadougou’s population is no exception. For example, in the Ouaga HDSS areas, nearly half of deaths are attributable to noncommunicable diseases [46].

The health care system is characterized by the fee for service and the lack of health insurance for the majority of the population [47]. In 2010, in the Ouaga HDSS areas, only half of adult individuals who experienced morbid episodes had recourse to medical treatment [48]. Chronic diseases management programs (such as screening and curative services) are not fully developed [47].

## Methods

### Data

This study was based on data from the 2010 Health Survey conducted at the Ouaga HDSS, which is a demographic surveillance system set up in 2008 by the Institut Supérieur des Sciences de la Population (ISSP) de l’Université de Ouagadougou. The Ouaga HDSS is a member of the International Network for the Demographic Evaluation of Populations and Their Health (INDEPTH), which is comprised of 48 similar sites in 20 countries of Africa, Asia, and Oceania. The Ouaga HDSS follows the population living in five neighborhoods (two formal and three informal) at the northern outskirts of Ouagadougou. In contrast to formal neighborhoods, the informal neighborhoods refer to the areas built without approval from municipal authorities based on the official description of the municipal registry. The Ouaga HDSS does not claim to be representative of the entire city of Ouagadougou, but of its outer limits, where the population is more vulnerable, younger, and most often born in rural areas [41].

The 2010 Health Survey data were collected between February and September 2010 from a sample of individuals residing in the Ouaga HDSS areas during the period immediately following the first population census. The survey was initially based on a sample of 1941 households drawn randomly and systematically using the Ouaga HDSS database as the sampling frame. In principle, all eligible individuals (questionnaires to be completed for those under 5 years of age and those 15 years or older) in these households were surveyed unless they refused or were absent. Altogether, 1699 households were questioned out of 1941 sampled, for a response rate of 87.5%.

The health survey was administered in face-to-face interviews by trained surveyors using Pocket PCs to promote consistency in collecting responses in the field. It included several types of health-related questionnaires. The health questionnaire for adults 15 years or older, which is the questionnaire used in the present analysis, consisted of eight sections addressing several health topics: descriptions of health status, functional limitations, accidents and violence, depression, physical activity, nutrition, alcohol, tobacco, access to services, anthropometric measures, and blood pressure measures. Information on age, education, marital status, and ethnicity of the respondents was obtained from the routine Ouaga HDSS data.

In all, 2351 persons 15 years or older were questioned. Of those, 156 observations (6.6%) were excluded from the present analysis because of missing data on SRH (dependent variable). Values for missing data (200 missing values) on all the independent variables were imputed by employing multiple imputation using chained equations (ICE) [49,50] to overcome the problem of missing observations in the multivariate analysis. ICE is a multiple imputation method well described in StataCorp [51]. Briefly, ICE iteratively fills in missing values in multiple variables by using chained equations, which are univariate imputation models, one for each imputation variable, with fully conditional specifications (FCS) for the prediction equations. All of the variables, except the one to be imputed, are included in the prediction equation. Based on the recommendations of van Buuren and colleagues [50], five different sets of data were imputed to reflect the uncertainty around the missing values.

The protocol research and the informed consent form were approved by the Ethics Committee of the Ministry of Health of Burkina Faso. Using this approved form, interviewers obtained informed consent for participation, in writing, from the respondents.

### Variables

#### Dependent variable: self-rated health

SRH was measured using a five-point scale (1 = very good, 2 = good, 3 = fair, 4 = poor, 5 = very poor) in response to the question, “In general, how would you rate your health today?” For the descriptive analyses, categories 1 and 2 (“very good” and “good”) were combined as “good,” and categories 4 and 5 (“poor” and “very poor”) as “poor” in order to examine the bivariate relationships between the independent variables and three levels of SRH. For the multivariate analyses, SRH was considered as a dichotomous variable: “fair” and “poor/very poor” were combined as “poor,” and this was compared with “very good/good”. The results of the multivariate analysis were not sensitive to this grouping of the responses, since the odds ratios produced by the ordinal models were nearly the same as those from the binary logistic regression which we present here.

#### The main independent variables

##### Chronic diseases

Chronic diseases were measured by asking respondents to indicate whether they had ever been told by a medical professional that they had one of the chronic diseases presented on a given list. The conditions listed included hypertension, diabetes, chronic bronchitis or asthma, angina pectoris, stroke, arthritis, gout, and stomach ulcer. A summative chronic conditions score (from 0 to 8) was calculated for each respondent and recoded into two categories: no condition (0) and one or more conditions (1).

##### Functional limitations

Functional limitations were assessed using the Short Set of Questions on Disability developed by the Washington Group on Disability Statistics (WG) [52]. Using a four-point scale (1 = no, no difficulty; 2 = yes, some difficulty; 3 = yes, a lot of difficulty; 4 = cannot do at all), this questionnaire consists of six questions on health-related difficulties in six core functional domains: vision, hearing, mobility, cognition, self-care, and communication. The WG questions reflect the advances made in conceptualizing disability and use the World Health Organization’s International Classification of Functioning, Disability and Health (ICF) [53] as a conceptual framework. In our analyses, the scores were coded in binary format (0 = no difficulty; 1 = any difficulty), and a summative functional limitations score (0 to 6) was calculated and recoded into three categories: no difficulty (0), one difficulty (1), and two or more difficulties (2).

##### Depression

The diagnosis of depression was assessed by diagnostic structured interviews based on the Major Depression Module of the Mini International Neuropsychiatric Interview (MINI) [54], a questionnaire with nine questions based on the criteria of the Diagnostic and Statistical Manual of Mental Disorders, Fourth Edition (DSM-IV) [55]. Using a yes–no response format, this questionnaire is made up of two initial questions posed to all respondents on any (1) decline in overall mood and (2) loss of interest in activities, which have persisted chronically over at least the previous two weeks; for respondents reporting one or both of these symptoms, there are a further seven questions on appetite, sleep, behavior, fatigue, self-esteem, concentration, and suicidal thoughts. These nine questions were presented in French or in Mooré, the primary local language spoken in Ouagadougou. The forward-backward translation method was used to standardize the process of translation across respondents. Based on the MINI cut-off criteria, individuals either presented a major depression disorder at the time of the survey (at least 5 symptoms, including at least one of the first two) or not.

Overall, approximately one-third (30.9%) of the persons in our sample declared themselves to be in fair or poor health; 21% reported having at least one chronic condition; 16% reported having one functional limitation and 12% reported having two or more limitations; 4.5% of those sampled presented a major depressive disorder (Table 1 and Table 2).

**Table 1:** **Descriptive statistics for chronic diseases, depression, and functional limitations by sex in adults (n = 2,195) in Ouagadougou**^**a**^**, Ouaga HDSS Health Survey, 2010**

	**Men**		**Women**		
	**n**	**% or mean (SD)**	**n**	**% or mean (SD)**	**p-value**^**b**^
**Hypertension**					
No	908	94.3	1085	90.7	.002
Yes	77	5.7	125	9.3	
**Diabetes**					
No	969	98.7	1195	98.8	.835
Yes	16	1.3	15	1.2	
**Bronchitis / Asthma**					
No	939	95.1	1166	95.7	.570
Yes	46	4.9	44	4.3	
**Angina pectoris**					
No	965	97.8	1141	93.2	<.001
Yes	20	2.2	69	6.8	
**Stroke**					
No	966	97.6	1164	95.2	.003
Yes	19	2.4	46	4.8	
**Arthritis / Rheumatism**					
No	969	98.2	1196	98.8	.214
Yes	16	1.8	16	1.2	
**Gout**					
No	978	99.4	1196	98.5	.064
Yes	7	0.6	14	1.5	
**Stomach ulcer**					
No	934	94.2	1109	91.0	.007
Yes	51	5.8	101	9.0	
**Number of chronic diseases**	985	0.25 (0.022)	1210	0.38 (0.031)	.001
**Depression**					
Not depressed	951	96.2	1157	95.0	.145
Depressed	34	3.8	53	5.0	
**Vision**					
No difficulty	790	86.4	939	84.2	.122
Any difficulty	195	13.6	271	15.8	
**Hearing**					
No difficulty	922	95.2	1103	93.4	.04
Any difficulty	63	4.8	107	6.6	
**Mobility**					
No difficulty	854	91.3	929	83.3	<.001
Any difficulty	131	8.7	281	16.7	
**Memory**					
No difficulty	869	90.8	964	84.1	<.001
Any difficulty	116	9.2	246	15.9	
**Self-care**					
No difficulty	957	98.2	1156	96.3	.006
Any difficulty	28	1.8	54	3.7	
**Communication**					
No difficulty	965	98.5	1182	98.2	.578
Any difficulty	20	1.5	28	1.8	
**Functional limitations score**					
No limitation	688	76.5	714	67.5	<.001
One limitation	169	15.1	229	16.8	
Two or more limitations	128	8.4	267	15.7	

^a^Proportions and means are weighted using sampling weights provided by the Ouaga HDSS Health Survey and take the clustering at household level into account. Absolute frequencies (n) are unweighted.

^b^p-value based on Chi-squared test for proportions differences or t-test for mean (standard deviation) differences between men and women.

**Table 2:** **Bivariate associations of measures of physical and mental health with self-rated health in adults (n = 2,195) in Ouagadougou**^**a**^**, Ouaga HDSS Health Survey, 2010**

	**n**	**Good**	**Fair**	**Poor**	
		**%**	**%**	**%**	**Chi2**
**Hypertension**					59.1
No	1993	71.2	23.1	5.7	*p* < 0.001
Yes	202	44.2	39.9	15.1	
**Diabetes**					10.1
No	2164	69.5	24.1	6.4	*p* = 0.012
Yes	31	41.1	47.4	11.5	
**Bronchitis / Asthma**					19.5
No	2105	70.0	23.8	6.2	*p* < 0.001
Yes	90	49.9	37.1	13.0	
**Angina pectoris**					29.5
No	2106	70.3	23.5	6.2	*p* < 0.001
Yes	89	45.2	42.0	12.8	
**Stroke**					30.3
No	2130	69.9	24.2	6.0	*p* < 0.001
Yes	65	49.2	30.8	20.0	
**Arthritis / Rheumatism**					13.0
No	2163	69.5	24.1	6.3	*p* = 0.004
Yes	32	41.4	41.8	16.9	
**Gout**					37.8
No	2174	69.6	24.2	6.2	*p* < 0.001
Yes	21	22.4	44.6	33.0	
**Stomach ulcer**					39.5
No	2043	70.9	22.9	6.2	*p* < 0.001
Yes	152	47.5	42.6	9.9	
**Number of chronic diseases**					124.0
No condition	1719	74.8	20.3	4.9	*p* < 0.001
One or more condition	476	48.3	39.4	12.3	
**Depression**					34.9
Not depressed	2108	70.1	24.0	5.9	*p* < 0.001
Depressed	87	48.0	33.0	19.0	
**Vision**					152.7
No difficulty	1729	73.6	22.0	4.4	*p* < 0.001
Any difficulty	466	43.3	38.0	18.7	
**Hearing**					79.5
No difficulty	2025	70.7	23.8	5.4	*p* < 0.001
Any difficulty	170	42.7	33.7	23.6	
**Mobility**					244.4
No difficulty	1783	74.2	22.0	3.9	*p* < 0.001
Any difficulty	412	35.6	40.5	24.0	
**Memory**					148.8
No difficulty	1833	73.0	22.5	4.5	*p* < 0.001
Any difficulty	362	42.5	37.3	20.2	
**Self-care**					189.9
No difficulty	2113	70.4	24.4	5.3	*p* < 0.001
Any difficulty	82	25.6	26.1	48.4	
**Communication**					71.2
No difficulty	2147	69.8	24.3	5.9	*p* < 0.001
Any difficulty	48	29.1	31.8	39.1	
**Functional limitations score**					322.6
No limitation	1402	77.9	19.3	2.8	*p* < 0.001
One limitation	398	56.0	35.7	8.3	
Two or more limitations	395	35.0	39.2	25.8	

^a^Proportions are weighted using sampling weights provided by the Ouaga HDSS Health Survey and take the clustering at household level into account. Absolute frequencies (n) are unweighted.

#### Other independent variables

##### Health-related factors

Four health-related factors were considered: body mass index (BMI), alcohol consumption, current tobacco use, and physical activity. In accordance with the WHO cut-off criteria, we categorized BMI into four conditions: underweight (BMI < 18.5), normal weight (18.5 ≤ BMI < 25), overweight (25 ≤ BMI < 30), and obesity (BMI ≥ 30). Alcohol consumption was measured by a single item from WHO’s Alcohol Use Disorders Identification Test (AUDIT) [56]. The item indicated the respondent’s frequency of consumption of alcohol (beer, wine, spirits, liquors, or other alcoholic drinks) over the previous 12 months using the responses: “never,” “once a month or less,” “two to four times per month,” “two or three times per week,” “four to six times per week,” and “daily.” Alcohol consumption was dichotomized: “daily,” “four to six times per week,” and “two or three times per week” were combined as (1), and “never,” “once a month or less,” and “two to four times per month” were combined as (0). Current tobacco use was a variable indicating whether the respondent smoked cigarettes, cigars, or pipes at the time of the survey. Physical activity was measured by a question asking each respondent to indicate the number of days in the prior seven in which he or she had to carry out physical exercise (strenuous physical labor, bicycling, walking) for at least 10 minutes.

##### Socio-demographic variables

The socio-demographic variables retained for this analysis were age at time of survey, marital status, ethnicity, and education.

#### Statistical analysis

We began with bivariate associations of SRH with chronic diseases, functional limitations, and depression. We then used logistic regression models to examine the associations between SRH and the three physical and mental health conditions (chronic diseases, functional limitations, and depression) as well as the other explanatory variables.

We estimated three types of logistic regression models. First, we evaluated the effect of each health condition separately as well as that of each of the other explanatory variables (gross effects model). Then, we simultaneously included in the same model the measures of chronic diseases, functional limitations, and depression, as well as the other independent variables to assess the net effect of each variable (net effects model). The final step of the analysis consisted of examining the variations in the relationship between SRH and physical and mental health measures according to sex, age, and education level. We thus fitted stratified logistic models by sex, age, and education levels. To test if sex, age, and education level modified the associations of SRH with chronic diseases, functional limitations, and depression, we fitted three pooled logistic regressions for SRH, which included interaction terms between the health condition and, respectively, sex, age, and education level. In all models, we calculated linearized standard errors [57] to take into account the correlated nature of responses from individuals in the same household.

All of the analyses, both descriptive and multivariate, used the available survey weights to take into account the sampling plan and nonrespondent households.

## Results

Table 2 presents the bivariate associations between SRH and the health measures considered. As it might be expected, fair or poor SRH were more often reported by individuals with chronic diseases, depression, or functional limitations, whereas good SRH was more often reported by those without such health problems. 51.7% of individuals with one or more chronic diseases versus 25.2% of those without chronic conditions reported fair or poor SRH. A similar pattern was found in relation to functional limitations, where a higher percentage of individuals with functional limitations than those without limitations reported fair or poor SRH. Most striking were differences in mobility, with nearly 65% of those with mobility problems reporting themselves as being in fair or poor health, compared with only 26% of those without mobility limitations. Likewise, depressed persons more often reported fair or poor SRH than nondepressed persons (52.0% versus 29.9%). Concerning our three covariates of interest (age, sex, and education), we found (results not shown) that the proportion of individuals who reported being in fair or poor health increased steadily with age (*p* < .001) was higher for women compared to men (*p* < .001) and was highest among those with no education (*p* < .001).

Table 3 presents the results of the logistic regression models for poor SRH. In the gross effects model, odds ratios greater than 1 on the health status measures indicate that having more chronic diseases, being depressed, and having a high functional limitations score increased the likelihood of perceiving one’s health status as poor. Likewise, being a woman, being older, and being separated, widowed, or divorced increased that likelihood. On the contrary, having a higher level of education, being single, smoking daily, and having more days that include physical activity all reduced that likelihood. Ethnicity, BMI, and alcohol consumption were all minimally or not at all associated with poor SRH.

**Table 3:** **Odds ratios for poor self-rated health in adults (n = 2,195) in Ouagadougou**^**a**^**, Ouaga HDSS Health Survey, 2010**

**Variables**	**Gross effects**	**Net effects**
	**OR (95% CI)**	**OR (95% CI)**
**Number of chronic diseases**		
No condition^b^		
One or more conditions	3.17 (2.47-4.07)***	2.26 (1.71-3.00)***
**Depression**		
Not depressed^b^		
Depressed	2.54 (1.58-4.08)***	1.18 (0.69-2.04)
**Functional limitations score**		
No limitation^b^		
One limitation	2.77 (2.13-3.61)***	1.91 (1.43-2.54)***
Two or more limitations	6.55 (4.85-8.85)***	2.95 (2.09-4.17)***
**Body mass index**		
Underweight (BMI <18.5)	1.16 (0.85-1.57)	0.98 (0.69-1.39)
Normal weight (18.5 ≤ BMI <25)^b^		
Overweight (25 ≤ BMI <30)	0.94 (0.72-1.22)	0.79 (0.59-1.06)
Obesity (BMI ≥30)	1.33 (0.87-2.05)	0.87 (0.53-1.41)
**Tobacco use**		
Nonsmoker / Occasional smoker^b^		
Daily smoker	0.50 (0.33-0.76)***	0.73 (0.45-1.18)
**Alcohol consumption**		
Never / 1 to 4 times per month^b^		
Daily / 2 to 6 times per week	1.41 (1.03-1.93)**	0.92 (0.65-1.30)
**Number of days of physical activity**	0.96 (0.92-0.99)**	0.97 (0.93-1.01)
**Sex**		
Male^b^		
Female	1.76 (1.44-2.14)***	1.46 (1.13-1.88)***
**Age group**		
15–24 years^b^		
25–34 years	1.18 (0.88-1.59)	1.08 (0.76-1.54)
35–49 years	1.62 (1.18-2.22)***	1.28 (0.87-1.88)
50–64 years	3.12 (2.29-4.26)***	2.00 (1.31-3.05)***
65 years or older	6.82 (4.60-10.12)***	2.90 (1.69-5.00)***
**Ethnicity**		
Mossi^b^		
Other	1.12 (0.77-1.64)	1.06 (0.71-1.58)
**Education**		
No education^b^		
Primary school	0.58 (0.43-0.77)***	0.88 (0.65-1.20)
Secondary school or higher	0.56 (0.43-0.74)***	1.07 (0.77-1.49)
**Marital status**		
Single	0.56 (0.43-0.73)***	0.87 (0.62-1.21)
Married^b^		
Separated / Widowed / Divorced	3.06 (2.23-4.16)***	1.39 (0.97-1.99)*

*p < 0.10; **p < 0.05; ***p < 0.01.

^a^The models are weighted using sampling weights provided by the Ouaga HDSS Health Survey and take the clustering at household level into account. ^b^Reference group.

“Gross effects” models include one explanatory variable. “Net effects” models include all explanatory variables.

In the net effects model, while their corresponding odds ratios were lower than in the gross effects model, the number of chronic diseases (OR = 2.26, 95% CI = [1.71-3.00] for those with one or more conditions), the functional limitations score (OR = 1.91, 95% CI = [1.43-2.54] for those with one limitation; OR = 2.95, 95% CI = [2.09-4.17] for those with two or more limitations), sex (OR = 1.46, 95% CI = [1.13-1.88] for women), and age (for example, OR = 2.90, 95% CI = [1.69-5.00] for adults 65 years or older) all remained significant predictors of SRH. On the other hand, the significance levels for depression, education level, and tobacco use subsided, suggesting that these factors were not significantly associated with SRH. The significance level for marital status also declined considerably.

In the last step of the analysis, we examined how the associations between SRH and measures of physical and mental health varied by sex, age, and education level. In the analyses stratified by sex (Table 4), the number of chronic diseases and the functional limitations score were significant predictors of SRH in both men and women, while depression had a similar effect across sexes. The last column in Table 4 provides the *p* values associated with interaction terms between sex and individual physical and mental conditions. The results showed that although women were more likely than men to report having many chronic diseases and functional limitations (See Table 1), there was no sex variation in the effects of measures of physical and mental health on poor SRH. Ancillary analyses explored the varying effects of a selection of particular chronic diseases (hypertension, bronchitis, angina, stroke, and stomach ulcer) across sex. We ruled out chronic diseases that were very rare in our sample (diabetes, arthritis, gout). The results showed that even if the associations of poor SRH with each of the chronic conditions (except for hypertension and stomach ulcer) were stronger for men than for women, no sex variation in these associations was found, as illustrated by the nonsignificant interaction effects (see Additional file 1). Nevertheless, these findings may be imprecisely estimated because of the few reports of many chronic diseases in our sample, as illustrated by some large confidence intervals (see Additional file 1).

**Table 4:** **Odds ratios for poor self-rated health, stratified by sex in adults (n = 2,195) in Ouagadougou**^**a**^**, Ouaga HDSS Health Survey, 2010**

	**Men (n = 985)**	**Women (n = 1 210)**	
**Variables**	**OR (95% CI)**	**OR (95% CI)**	**Interaction test**
**Number of chronic diseases**			
No condition^b^			
One or more conditions	2.37 (1.57-3.57)***	2.16 (1.52-3.08)***	*p* = 0.855
**Depression**			
Not depressed^b^			
Depressed	1.14 (0.48-2.73)	1.23 (0.63-2.39)	*p* = 0.810
**Functional limitations score**			
No limitation^b^			
One limitation	1.81 (1.17-2.81)***	2.00 (1.37-2.92)***	*p* = 0.298
Two or more limitations	2.74 (1.60-4.70)***	3.09 (2.02-4.73)***	*p* = 0.958

*p < 0.10; **p < 0.05; ***p < 0.01.

^a^The models are weighted using sampling weights provided by the Ouaga HDSS Health Survey and take the clustering at household level into account. The models are adjusted for age, ethnicity, marital status, education level, body mass index, alcohol consumption, tobacco use, and physical activity.

^b^Reference group.

In the analyses stratified by age groups (Table 5), the functional limitations score was a significant predictor of SRH in all three of the age groups considered (15–34 years, 35–59 years, and 60 years or older), and the number of chronic diseases in 15–34 years and 35–59 years, but depression was not significantly associated with SRH in any age group. The last column in Table 5 provides the *p* values associated with interaction terms between age and individual physical and mental conditions. The results show that the probability of people with poor SRH in the presence of two or more functional limitations increased more for older individuals (60 years or older) than for younger individuals (15–34 years) (interaction test *p* = 0.019). Similarly, having two or more functional limitations was associated more strongly with poor SRH in middle-aged adults (35–59 years) than in younger adults (interaction test *p* = 0.026). Thus, the effect of functional limitations on poor SRH becomes stronger with age. However, although the test for interaction was not significant, chronic diseases were associated less strongly with poor SRH in older adults than in younger and middle-aged adults. By exploring the varying effects of particular chronic diseases across age groups, the results suggest three distinct patterns (see Additional file 1). For three conditions (hypertension, bronchitis, and stroke), the odds ratios increased in size until middle age (35–59 years) and then decreased strongly thereafter. While for angina the odds ratios decreased more consistently, for stomach ulcer, the odds ratios increased with age.

**Table 5:** **Odds ratios for poor self-rated health, stratified by age in adults (n = 2,195) in Ouagadougou**^**a**^**, Ouaga HDSS Health Survey, 2010**

	**15-34 years (n = 1,031)**	**35-59 years (n = 718)**	**60 years or older (n = 446)**	
**Variables**	**OR (95% CI)**	**OR (95% CI)**	**OR (95% CI)**	**Interaction test**
**Number of chronic diseases**				
No condition^b^				
One or more conditions	2.20 (1.45-3.34)***	2.97 (1.88-4.69)***	1.65 (0.85-3.22)	*p* = 0.152
**Depression**				
Not depressed^b^				
Depressed	0.95 (0.46-1.96)	1.72 (0.66-4.47)	1.18 (0.22-6.35)	*p* = 0.154
**Functional limitations score**				
No limitation^b^				
One limitation	1.67 (1.09-2.55)**	2.24 (1.40-3.59)***	2.56 (1.25-5.26)**	*p* = 0.026
Two or more limitations	1.82 (0.89-3.73)	3.81 (2.25-6.45)***	4.80 (2.33-9.89)***	*p* = 0.019

*p < 0.10; **p < 0.05; ***p < 0.01.

^a^The models are weighted using sampling weights provided by the Ouaga HDSS Health Survey and take the clustering at household level into account. The models are adjusted for sex, ethnicity, marital status, education level, body mass index, alcohol consumption, tobacco use, and physical activity.

^b^Reference group.

In the analyses stratified by education level (Table 6), the number of chronic diseases was a significant predictor of SRH for both noneducated and educated persons, while the functional limitations score was significantly associated with SRH only for noneducated persons. By repeating the analysis without including chronic diseases in the model among educated persons, we found (results not shown) that the effect of functional limitations on poor SRH became statistically significant, suggesting that chronic conditions are important confounders in the relationship between SRH and functional limitations in that group. No significant effect was observed for depression regardless of the education level considered. The last column in Table 6 provides the *p* values associated with interaction terms between education level and individual physical and mental conditions. The results show that education level modifies the effect of functional limitations on SRH, while the influence of chronic diseases is the same regardless of education level. For example, having two or more functional limitations was associated more strongly with poor SRH in noneducated persons than in educated persons (interaction test *p* = 0.001). Ancillary analyses exploring the varying effects of particular chronic diseases on poor SRH across education level showed that although the interaction terms were not statistically significant, for all but two of the chronic conditions (bronchitis and stomach ulcer), the strongest odds ratio was found among educated persons (see Additional file 1).

**Table 6:** **Odds ratios for poor self-rated health, stratified by education level in adults (n = 2,077) in Ouagadougou**^**a**^**, Ouaga HDSS Health Survey, 2010**

	**Not educated (n = 1,228)**	**Educated**^**c **^**(n = 849)**	
**Variables**	**OR (95% CI)**	**OR (95% CI)**	**Interaction test**
**Number of chronic diseases**			
No condition^b^			
One or more conditions	2.07 (1.40-3.04)***	2.93 (1.89-4.55)***	*p* = 0.899
**Depression**			
Not depressed^b^			
Depressed	1.15 (0.51-2.61)	1.27 (0.62-2.64)	*p* = 0.364
**Functional limitations score**			
No limitation^b^			
One limitation	2.71 (1.86-3.96)***	1.39 (0.86-2.23)	*p* = 0.033
Two or more limitations	4.42 (2.93-6.67)***	1.21 (0.61-2.41)	*p* = 0.001

*p < 0.10; **p < 0.05; ***p < 0.01

^a^The models are weighted using sampling weights provided by the Ouaga HDSS Health Survey and take the clustering at household level into account. The models are adjusted for sex, age, ethnicity, marital status, body mass index, alcohol consumption, tobacco use, and physical activity.

^b^Reference group.

^c^Educated people include persons with primary, secondary, or more education.

## Discussion

The objective of this study was to examine the associations between SRH and the dimensions of physical and mental health in Ouagadougou, Burkina Faso, and how these associations varied by sex, age, and education level. Our results showed positive associations between poor SRH and the presence of chronic diseases and functional limitations, with the latter being stronger than the former. On the contrary, the relationship between SRH and depression was shown to be nonsignificant after controlling for the other factors. These results suggest that, in the context of this study, SRH reflects aspects of physical health more than of mental health.

Our results highlighted women’s disadvantage in SRH. In our sample, this disadvantage for women persisted even after adjusting for other variables. The same trend was observed for age, with older persons being more likely to report being in poor health than younger adults. For education level, adding other variables completely eliminated its effect.

In our analyses, when participants were compared at the same levels of health problems (chronic diseases, functional limitations, and depression), men and women appeared to assess their health in a similar way. Some previous studies also obtained this result [28,29] and suggested that men and women seemed to use the same criteria when assessing their health status. Thus, our results, which show no sex variation in the effect of health problems on SRH, do not support the contentions of some researchers [27] that women generally see themselves as being in poor health more than men because they have higher health expectations. Rather, these results tend toward the interpretation that women really are in worse health [28]. For example, most of the functional limitations included in our analysis are more prevalent in women than in men (see Table 1). These results could be explained partly by the fact that in our sample the poorest women were overwhelmed with certain economic activities (for example, selling vegetables and condiments and activities related to aesthetics), domestic tasks (cooking over a fire and caring for a numerous offspring), and other activities often physically painful (for example, gathering pebbles and sand, brick making, and water transport); the less poor women were often housewives who were overweight and engaged in fewer physical and intellectual activities [42]. Additionally, the differences between men and women in certain objective health measures such as physical performance indicators, which are not measured in this study, may significantly contribute to the female disadvantage in SRH. For example, some studies conducted in the developing world have showed that women are less able than men to perform several physical tasks related to balance, gait, and lower- and upper-extremity movement [58].

In this study, the effect of functional limitations on SRH intensified with age. However, although the test for interaction was not significant, chronic diseases had a lower impact on poor SRH in older persons than in middle-aged persons. The effect of depression was similar across all ages. Our results on the modifying effect of age on the relationship between SRH and chronic diseases appear to agree with those obtained by Mäntyselkä and colleagues [59] in Finland who showed that chronic diseases were much less strongly associated with poor SRH in older persons (45–74 years) than in younger persons (15–44 years). They are also in keeping with those from Schnittker [11] that found that the effect of chronic diseases on SRH diminished with age, using American data on persons 25 years or older. However, the results of the latter study [11] indicating that the effect of functional limitations also declined with age were contrary to our own findings.

Schnittker [11] suggested that the results of his study related to the association between SRH and chronic diseases and functional limitations were consistent with social comparison theory. Given that our results do not go entirely in the same direction as Schnittker’s, we therefore offer alternative explanatory hypotheses for our study. In our sample, the fact that the association between SRH and functional limitations increased with age and the observation that the relationship with chronic diseases appeared to decrease with age, suggest that older persons tend to focus more on their functional limitations, probably because their ability to circumvent or compensate for disabilities diminishes with age; as for younger and middle-aged adults, they seem to concentrate more on chronic diseases. Jylhä [8] has already indicated that among younger adults, chronic conditions or functional limitations are used to substantiate negative health assessments. Here, our data suggest that younger adults and especially middle-aged adults seem to base their self-assessment of health on chronic diseases.

We also found that education level modified the effect of functional limitations on SRH (i.e., the effect was stronger in noneducated persons) but not the effects of chronic diseases or depression. Our results were very different from those of the recent works [30-32] that found that the association between SRH and functional limitations and chronic diseases was stronger in more educated individuals. Basing themselves on social comparison theory, these authors explained their results in terms of a health expectations gap: those who are more educated have higher expectations than those who are less educated, and therefore their poor SRH affects them more strongly and negatively given the same health problems [32]. Here, our results on variations in the relationship between SRH and functional limitations according to education level do not support the social comparison hypothesis. Thus, a plausible hypothesis to explain our results would be that noneducated persons refer to functional limitations as a framework for their assessment, probably because physical integrity is much more important for these people who depend on it for their livelihood.

The present study has certain limitations that should be noted. First, because the data are cross-sectional, our ability to understand the direction of the relationships among the variables is limited. Since the question on SRH was asked before those on physical and mental health, we can exclude that the latter influenced the evaluation expressed in the former. Second, all measures of physical and mental health considered were self-reported, with all of the limitations that this method entails, mostly the underreporting of chronic diseases in socially disadvantaged groups (particularly noneducated persons). This could have the effect of underestimating the effect of chronic diseases on SRH for these groups. Additionally, it is possible that some of the effects of functional limitations that are found here are attributable to different ways in which different subpopulations delineated by sex, age, or education level answer questions.

## Conclusions

Research has suggested that SRH reflects a wide range of physical and mental dimensions of health that contribute to the overall self-assessment of health status [8]. In this study, we found that SRH was strongly correlated with chronic diseases and functional limitations, but it was not linked to depression, which would tend to suggest that SRH is more a reflection of the physical aspects of health in this setting.

Up to now, studies conducted in Africa have used SRH to assess the health of populations based on a series of demographic and socio-economic characteristics. However, almost none of them have sought to know what aspects of health were being considered and what factors affected how these aspects were taken into account in SRH. In this respect, the present study makes a significant contribution. First, it shows physical health aspects (functional limitations and chronic diseases) to be the potential elements of health that make up SRH. Second, our study highlights the heterogeneity of reports of SRH in relation to age and education level. Indeed, our results indicate that the association between poor SRH and functional limitations increases with age and decreases with education level. On the other hand, the association between poor SRH and chronic diseases appears to diminish with age. No sex variation in the effect of health problems on SRH is found. These findings suggest that age and education level affect the way in which the components of health are taken into account in SRH. In-depth studies are needed to understand why and how these groups do so.

## Abbreviations

BMI: Body mass index; ICE: Imputation using chained equations; MINI: Mini International Neuropsychiatric Interview; Ouaga HDSS: Ouagadougou Health and Demographic Surveillance System; SRH: Self-rated health; WG: Washington Group on Disability Statistics.

## Competing interests

The authors declare that they have no competing interests.

## Authors’ contributions

YO conceived of the study, performed the statistical analyses, and wrote the first draft. SB contributed to the conception, analysis, and interpretation of results and helped to draft the manuscript. CR managed and coordinated the data collection, contributed to the interpretation of results, and was involved in revising the manuscript. MVZ contributed to the conception, analysis, and interpretation of results and helped to draft the manuscript. All authors read and approved the final manuscript.

## Supplementary Material

Additional file 1: Table S1 Odds ratios from 10 logistic regressions of poor self-rated health on chronic diseases, stratified by sex in adults (n = 2,195) in Ouagadougou, Ouaga HDSS Health Survey, 2010. **Table S2**. Odds ratios from 14 logistic regressions of poor self-rated health on chronic diseases, stratified by age groups in adults (n = 2,195) in Ouagadougou, Ouaga HDSS Health Survey, 2010. **Table S3**. Odds ratios from 10 logistic regressions of poor self-rated health on chronic diseases, stratified by education level in adults (n = 2,077) in Ouagadougou, Ouaga HDSS Health Survey, 2010. Click here for file
