# Supplementary material for: The components of self-rated health among adults in Ouagadougou, Burkina Faso
Source: Popul Health Metr. 2013 Aug 8;11:15. doi: 10.1186/1478-7954-11-15 (PMC3750468; doi:10.1186/1478-7954-11-15)
Supplement: Additional file 1: Table S1 — Odds ratios from 10 logistic regressions of poor self-rated health on chronic diseases, stratified by sex in adults (n = 2,195) in Ouagadougou, Ouaga HDSS Health Survey, 2010. Table S2. Odds ratios from 14 logistic regressions of poor self-rated health on chronic diseases, stratified by age groups in adults (n = 2,195) in Ouagadougou, Ouaga HDSS Health Survey, 2010. Table S3. Odds ratios from 10 logistic regressions of poor self-rated health on chronic diseases, stratified by education level in adults (n = 2,077) in Ouagadougou, Ouaga HDSS Health Survey, 2010. [file 1478-7954-11-15-S1.docx]

**Table S1.** **Odds ratios from 10 logistic regressions of poor self-rated health on chronic diseases, stratified by sex in adults (n = 2 195) in Ouagadougou^a^, Ouaga HDSS Health Survey, 2010**

|  | Men (n=985) | Women (n=1 210) |  |
| --- | --- | --- | --- |
| Variables | OR (95% CI) | OR (95% CI) | Interaction test |
| **Hypertension** |  |  |  |
| No^b^ |  |  |  |
| Yes | 1.86 (0.94-3.66)* | 2.36 (1.45-3.83)*** | *p* = 0.648 |
| **Bronchitis** |  |  |  |
| No^b^ |  |  |  |
| Yes | 3.79 (1.96-7.33)*** | 2.19 (1.16-4.13)** | *p* = 0.255 |
| **Angina** |  |  |  |
| No^b^ |  |  |  |
| Yes | 3.60 (1.26-10.27)** | 2.70 (1.53-4.76)*** | *p* = 0.762 |
| **Stroke** |  |  |  |
| No^b^ |  |  |  |
| Yes | 4.06 (1.56-10.54)*** | 2.46 (1.23-4.93)*** | *p* = 0.530 |
| **Stomach ulcer** |  |  |  |
| No^b^ |  |  |  |
| Yes | 2.55 (1.37-4.73)*** | 3.33 (2.11-5.26)*** | *p* = 0.402 |

**p* < 0.10; ***p* < 0.05; ****p* < 0.01

^a^The models are weighted using sampling weights provided by the Ouaga HDSS Health Survey, and take the clustering at household level into account. All models are adjusted for age, ethnicity, marital status, education level, body mass index, alcohol consumption, tobacco use, and physical activity.

^b^Reference group.

**Table S2. Odds ratios from 14 logistic regressions of poor self-rated health on chronic diseases, stratified by age groups in adults (n = 2 195) in Ouagadougou^a^, Ouaga HDSS Health Survey, 2010**

|  | 15-34 years (n=1 031) | 35-59 years (n=718) | 60 years and older (n=446) |  |
| --- | --- | --- | --- | --- |
| Variables | OR (95% CI) | OR (95% CI) | OR (95% CI) | Interaction test |
| **Hypertension** |  |  |  |  |
| No^b^ |  |  |  |  |
| Yes | 1.59 (0.70-3.62) | 3.55 (2.04-6.15)*** | 1.54 (0.77-3.08) | *p* = 0.141 |
| **Bronchitis** |  |  |  |  |
| No^b^ |  |  |  |  |
| Yes | 1.98 (1.00-3.92)** | 5.87 (2.46-13.97)*** | 1.73 (0.39-7.74) | *p* = 0.075 |
| **Angina** |  |  |  |  |
| No^b^ |  |  |  |  |
| Yes | 3.50 (1.88-6.53)*** | 2.08 (0.90-4.83)* | 1.91 (0.31-11.67) | *p* = 0.338 |
| **Stroke** |  |  |  |  |
| No^b^ |  |  |  |  |
| Yes | 1.95 (1.01-3.78)** | 5.48 (1.78-16.91)*** | NA | *p* = 0.087 |
| **Stomach ulcer** |  |  |  |  |
| No^b^ |  |  |  |  |
| Yes | 2.72 (1.58-4.67)*** | 2.62 (1.46-4.70)*** | 5.50 (1.72-17.61)*** | *p* = 0.248 |

**p* < 0.10; ***p* < 0.05; ****p* < 0.01

^a^The models are weighted using sampling weights provided by the Ouaga HDSS Health Survey, and take the clustering at household level into account. All models are adjusted for sex, ethnicity, marital status, education level, body mass index, alcohol consumption, tobacco use, and physical activity.

^b^Reference group.

NA: Not applicable. The model could not be estimated for stroke among elderly persons (60 years or older) because of small sample size.

**Table S3. Odds ratios from 10 logistic regressions of poor self-rated health on chronic diseases, stratified by education level in adults (n = 2 077) in Ouagadougou^a^, Ouaga HDSS Health Survey, 2010**

|  | Non-educated (n=1228) | Educated (n=849) |  |
| --- | --- | --- | --- |
| Variables | OR (95% CI) | OR (95% CI) | Interaction test |
| **Hypertension** |  |  |  |
| No^b^ |  |  |  |
| Yes | 2.16 (1.34-3.49)*** | 2.70 (1.40-5.22)*** | *p* = 0.988 |
| **Bronchitis** |  |  |  |
| No^b^ |  |  |  |
| Yes | 3.08 (1.64-5.81)*** | 2.30 (1.07-4.93)** | *p* = 0.626 |
| **Angina** |  |  |  |
| No^b^ |  |  |  |
| Yes | 2.54 (1.34-4.80)*** | 4.85 (1.89-12.45)*** | *p* = 0.308 |
| **Stroke** |  |  |  |
| No^b^ |  |  |  |
| Yes | 2.52 (1.23-5.20)** | 2.95 (1.17-7.41)** | *p* = 0.816 |
| **Stomach ulcer** | |  |  |
| No^b^ |  |  |  |
| Yes | 3.05 (1.79-5.21)*** | 2.68 (1.54-4.66)*** | *p* = 0.731 |

**p* < 0.10; ***p* < 0.05; ****p* < 0.01

^a^The models are weighted using sampling weights provided by the Ouaga HDSS Health Survey, and take the clustering at household level into account. All models are adjusted for sex, age, ethnicity, marital status, body mass index, alcohol consumption, tobacco use, and physical activity.

^b^Reference group.
